# Apolipoprotein B and Lipid Profile in Italian Children and Adolescents

**DOI:** 10.3390/jcdd11020044

**Published:** 2024-01-29

**Authors:** Francesco Martino, Tarcisio Niglio, Eliana Martino, Vincenzo Paravati, Luisa de Sanctis, Ornella Guardamagna

**Affiliations:** 1 Department of Internal Medicine, Anesthesiology and Cardiovascular Science, Sapienza University of Rome, I-00161 Rome, Italy; elianamartino@inwind.it (E.M.); vincenzo.paravati@uniroma1.it (V.P.); 2 Istituto Superiore di Sanità, I-00161 Roma, Italy; tarcisio.niglio@iss.it; 3 Department of Public Health and Pediatric Sciences, University of Turin, I-10126 Turin, Italy; luisa.desanctis@unito.it (L.d.S.);

**Keywords:** apolipoproteins B, lipid metabolism disorders, dyslipidemias, pediatrics

## Abstract

Rationale. The prevention of cardiovascular (CV) disease is mandatory from childhood onwards. Among biochemical markers related to the clinical cardiovascular outcome, LDL cholesterol (LDL-C), non-HDL-C and apolipoprotein B (ApoB) are recognized as main target parameters. Emphasis on ApoB concentrations is growing, as representative of any class of atherogenic lipoprotein. This consideration allows checking of subjects under 18 years of age when the CV risk occurs. The aim of this study is to evaluate ApoB levels in a sample of Italian hyperlipidemic children and adolescents, and their siblings, to test any relationship with their lipid profile. Methods. A retrospective study, including 1877 children and adolescents (aged 0–18 years), was performed. Clinical and biochemical data were selected from a database, including the lipid profile, ApoB analysis and anthropometric parameters of any proband. Participants had been checked as potentially hyperlipidemia affected, the suspicion raised by familial CV risk or because the dyslipidemia was already known. Data from the first visit at the University Hospitals in Rome and Turin were collected. Patients affected by secondary hyperlipidemia or obesity were excluded. Blood test analysis was performed in fasting conditions by automated commercial kits. Participants were classified according to gender, age (stratified in subgroups: 0–5, 6–10, 11–14, and 15–18 years old) and anthropometric parameters, referred to as weight in Kg and height in cm, and BMI calculated. Lipid profile results were stratified in relation to acceptable, borderline, or increased levels, as indicated by NCEP, and any potential relation with ApoB established. Statistics were performed by Epi-Info 7 programs to evaluate the variance analysis. Either parent could sign the informed consent. Results. Among the whole sample n.1010 and n.867 participants were females and males, respectively. TC values acceptable (≤170 mg/dL), borderline (171–200 mg/dL) and elevated (≥201 mg/dL) were found in 411 (22%), 585 (31%) and 881 (47%) participants, respectively. The LDL-C cut-off considered was 110 mg/dL (90° percentile). Mean ApoB progressively increased from 65 to 110 mg/dL according to TC levels and resulted in significant correlation when any age subgroup and gender was considered. The highest ApoB values, TC and LDL-C related, were found in the youngest subgroup, regardless of gender. Conclusion. ApoB results increase progressively and in parallel with TC and LDL-C and represent a further parameter to distinguish between normal and hyperlipidemic subjects. Serum levels are close to 70 mg/dL and to 100 mg/dL in the former and latter group, respectively.

## 1. Introduction

Atherosclerosis is a chronic degenerative process starting from childhood in the form of lipid streaks. These lesions are characterized by an abnormal, although reversible, accumulation of oxidized lipoprotein particles in the inner layer of the arterial wall. The consequence is narrowing of arteries, leading to the atheromatous plaque development. Lipid streaks represent the first step in the atherosclerotic process that progresses asymptomatically for decades and can cause ischemic syndromes if advanced to symptomatic stages [1].

Coronary artery disease (CAD), the leading cause of mortality and morbidity worldwide, represents the final stage of the dynamic atherosclerotic process, affecting the wall of medium and large size arteries [2]. The prolonged exposure to increased serum lipid levels and their cumulative effects are related to the severity of atherosclerosis [3,4].

Evidence supports the hypothesis that apolipoprotein B (ApoB), trapped in the thickness of the vascular wall, represents the driver in plaque development. This mechanism relates to the number of ApoB particles within the arterial lumen, which contribute to the progression of atherosclerosis, then to the risk of cardiovascular (CV) events. Therefore, the degree of each atherosclerotic plaque is likely to be proportional to the cumulative exposure to ApoB-containing lipoproteins [5].

Lipoproteins containing ApoB, synthetized by the liver, include VLDL, IDL, and LDL. These are characterized by one molecule of ApoB, phospholipids, and different contents of triglycerides and cholesterol: the former is mainly represented in VLDL, the latter in LDL. The ApoB particle size, therefore, varies significantly according to the lipid content [6,7]. Phospholipids, with ApoB as a further component when oxidized, are important proatherogenic factors [8].

TG-rich, cholesterol-rich and remnant lipoproteins, as all ApoB carrying lipoproteins, are equally atherogenic [9].

To estimate the CV risk, the assessment of ApoB or non-HDL-C shows high correlation with CAD risk, especially when LDL-C appears to be within the normal range [10]. ApoB is an independent risk factor for CAD, widely recognized, and neither LDL-C nor non-HDL-C are as accurate as ApoB in evaluating the cardiovascular risk [11]. Despite the advantage of ApoB and/or non-HDL-C in the accurate estimation of the risk of atherosclerotic CV disease, this view is not commonly shared. Controversies are still current [12,13] and LDL-C is, so far, commonly applied in practice [13,14,15,16]. In any case, it should be underlined that guidelines from the European Society of Cardiology and European Atherosclerosis Society (ESC/EAS), issued in 2019, suggest that ApoB is the best tool to assess CV risk, and in terms of adequacy for lipid-lowering treatment, compared to LDL-C or non-HDL-C [17].

Only a few studies have been conducted in children, but it has been shown that ApoB levels predict subclinical atherosclerosis in adulthood better than cholesterol levels [18]. Furthermore, in a large study conducted between 1999 and 2016 in young Americans, aged 6–19 years, a positive trend in lipid and ApoB levels was observed, suggesting the benefit of maintaining a low level of ApoB-containing lipoproteins throughout life [19]. 

Having established the growing value of ApoB detection in hyperlipidemia, we performed the present study to assess ApoB serum concentrations in Italian hyperlipidemic children and adolescents and their siblings. A further aim was to establish any potential relationship between ApoB and serum lipid parameters, then to distinguish normal, borderline, and pathological results, to evaluate ApoB as useful in practice to improve hyperlipidemia diagnosis.

## 2. Methods

A retrospective study was performed on a medical database generated from data available at the Lipid Clinic Research Centre (Department of Pediatrics, “Sapienza” University of Rome, Italy) and at the Dyslipidemia and Cardiovascular Prevention Unit (OIRM-Department of Health Science and Pediatrics, University of Turin, Italy). Each center included participant data from files obtained in the period 2002–2022 according to common inclusion and exclusion parameters and biochemical tests applied.

### 2.1. Subjects

Among a total of 49,321 subjects submitted to lipid laboratory tests, 1877 were selected and enrolled, showing all the required parameters including age 0–18 years, height, and weight measurement available, and blood analysis (total cholesterol TC, LDL-C, HDL-C, TG, ApoB).

Subjects affected by chronic disorders (including hematopoietic, cancer, renal, endocrine, and liver illness), undergoing long-lasting therapy (with retroviral, anticonvulsant, corticosteroids or other drugs affecting the lipid metabolism), or obese were excluded from the study.

All enrolled children had been medically examined. The authors collected the physiological and pathological anamnesis, together with anthropometric measurements. The weight and height were measured: body weight was measured in light clothes and without shoes and was approximated to the nearest 0.1 kg on a mobile digital scale (Seca, Hamburg, Germany), and height was measured to the nearest 0.1 cm using a wall mounted stadiometer (Seca, Hamburg, Germany) and BMI calculated (weight-kg/height-mt^2^). Results were considered acceptable when between the 25°–90° percentiles, according to Cacciari [20]. The 12 h fasting blood sample was drawn to analyze the lipid profile and ApoB. Biochemical analyses were performed by automated method applying commercial kits (Roche). LDL-C was calculated by the Friedewald equation: TC- (HDL-C)—TG/5. Lipoprotein values were divided in relation to values considered acceptable, borderline, or elevated [21].

Each enrolled child’s legal guardian included in this protocol had already signed a written informed consent document in respect of EU Regulation n.679/2016.

### 2.2. Statistical Analysis

Data were collected into a Microsoft Access 2017 database and analyzed by Epi-Info 7 programs (CDC and NIH, 2021 Italian version 7.2.5.0). Statistical analysis estimated frequencies, descriptive statistics, and statistical significance in group differences. Statistical significance “between” and “within” groups was calculated via continuous variables. An analysis of variance (one-way ANOVA) was performed to test the equality of mean values between groups for continuous variables, including Bonferroni, Kruskal–Wallis, and Newman–Keuls pairwise mean ± standard deviation (S.D.) comparison tests. Chi-square Yates corrected test was used for non-continuous variables via Statcalc and Analysis programs. A p level less than 0.05 was considered significant, and 95% confidence intervals were also calculated.

## 3. Results

The present study was performed on a sample of 1877 children (1010 female plus 867 male), including hyperlipidemic and normal subjects (Table 1). Anthropometric mean values for height, weight, and BMI are age/gender appropriate. Mean data (±S.D.) are listed and stratified by gender and age (Table 1).

ApoB average levels are different when distinguished by gender, age, and serum lipid parameters. These differences are statistically significant. Table 2, Table 3, Table 4 and Table 5 refer to TC, LDL-C, HDL-C and TG, respectively.

A positive and significant correlation is detected between any blood lipid parameters considered (TC, LDL-C, HDL-C, TG; mean ± S.D.) and ApoB (mean ± S.D.). The same result is obtained when gender is considered and age is stratified according to the Italian scholastic period: aged 1–5 (pre-scholar), aged 6–10 (primary school), aged 11–13 (secondary school), aged 14–18 (high school) (Table 2, Table 3, Table 4 and Table 5).

The above-mentioned differences and correlations of ApoB are more significant if they relate to TC and LDL-C rather than HDL-C and TG.

## 4. Discussion

The ApoB analysis is relevant for improvement of CV risk detection, as ApoB evidence demonstrates. Serum analysis has been considered in children since the 1990s, formerly as a candidate marker to screen for FH affected subjects [22,23], then to distinguish other dyslipidemias in subjects carrying ApoB exceeding 100 mg/dL levels [24].

It should be underlined that ApoB data reports and papers which are related to children are very scanty. Methodological approaches and potential ethnic variability are critical. To establish ApoB serum variations and their potential relationship with anthropological parameters and serum lipid data could improve clinical practice for early detection of subjects at CV risk. The present study represents the first wider approach to checking for ApoB levels in hyperlipidemic Italian children from different areas of the country: north, center and south.

Our results clearly indicate that ApoB serum levels increase progressively with TC, LDL-C and TG and inversely with HDL-C. These changes are significant and demonstrate the association between serum ApoB levels and any serum lipid parameter here considered. Inside each gender and age subgroup, this association is further confirmed as significant.

Mean ApoB serum concentrations correspond to 65 mg/dL, 80 mg/dL and 110 mg/dl when TC levels are ≤170 mg/dL, within the range 171–200 mg/dL or exceed 201 mg/dL, respectively. The above-mentioned TC concentrations are considered in the order, normal, acceptable, and high, as established by the Expert Panel on Blood Cholesterol levels in Children and Adolescents [25]. Considering LDL-C ≤ 110 mg/dL as an acceptable level, the corresponding mean ApoB here detected is 68 mg/dL. On this basis, we should assume that mean ApoB values between 65–68 mg/dL could be considered acceptable, as TC and LDL-C concentrations are. These results agree once more with the data of the Expert Panel on Blood Cholesterol levels in Children and Adolescents, which establish the normal ApoB cut-off level ≤ 90 mg/dL as acceptable [25]. 

A study was conducted on Japanese children, aged 8.0–9.7 years, to check for ApoB levels in healthy and familial combined hyperlipidemia (FCHL) affected subjects. ApoB levels were 63 mg/dL and 126 mg/dL, respectively, the authors concluded that it was relevant to add ApoB analysis to improve the diagnosis of FCHL [26]. Similar data were observed in Swedish school children, 10–12 years old, from the general population. Mean ApoB levels were 68 mg/dL in males and 75 mg/dL in females, showing a normal lipid profile. FH or FCHL affected children demonstrated serum ApoB concentrations exceeding the 95° percentile, the mean values corresponding to 98 mg/dL and 107 mg/dL in males and females, respectively [24]. A further study conducted in young adults from The Netherland (n.194), 18–20 years old, showed average ApoB levels of 75 mg/dL in healthy subjects, but increasing to 112 mg/dL when measured in FCHL participants, also BMI related [27]. The common finding that emerges from these studies, including the present one, is the concordance of ApoB levels between 63–75 mg/dL in healthy participants. This is an interesting and not negligible result, as different countries were included, and we can consider that the number of Italian subjects is not negligible. In addition, considering hyperlipidemic children and adolescents, the ApoB values show a wider spectrum, but data are commonly close to 100 mg/dL or exceed it. In the present study, TC and LDL-C stratification is according to percentile cut-off parameters [21,25]. The latter, applied here, although established in the USA, are widely used as representative of Caucasian ethnic groups, Italian included. The highest mean ApoB serum values, corresponding to TC ≥ 201 mg/dL, are shown in the subgroup 1–5 years old when compared to other age clusters. The same observation of ApoB concentration and progressive decrease provokes age-related concerns as to whether it should be attributed to the random occurrence of higher TC and LDL-C serum levels inside the subgroup 1–5 years old. Alternatively, a physiological decrease with age should not be excluded. Yearly ApoB changes, including increase or decrease, have been demonstrated in young adulthood through life, but no data are available in children [28]. This is an important point, requiring further tests to confirm and understand this trend.

Patients recruited vary in age, from childhood through adolescence to early adulthood, and the total number of subjects included is well representative of both genders. The participant numbers increase progressively with age, peaking at 8–9 years in females and similarly in males, through slightly later in age. This trend is representative of CV prevention needs. Furthermore, it confirms the actual and improved awareness of GPs, pediatricians, and cardiologists of the need for early diagnosis, and the consciousness of the need to apply the primary hyperlipidemia screening program in children from families who have experienced CV events. These include primarily monogenic and polygenic disorders. ApoB concentration has been considered an important diagnostic marker of FCHL as increased values are detectable earlier than other lipid biochemical changes occur. In selecting a candidate proband inside a family showing a cluster of CV risks, including metabolic syndrome pattern, the consideration to check for ApoB level should be beneficial. The precocity of the diagnostic suspicion addresses efforts to improve lifestyle, as the environment impacts on a healthy outcome, and to plan follow-up.

Anthropometric parameters confirm that weight and height, and thus BMI, were between the 25°–75° percentiles [21], while just a limited number of participants (25/1877) had a BMI exceeding the 90° percentile, but below the 95° percentile, and no obese participants was considered, as established in the recruiting criteria. This is an important point addressing dyslipidemia mainly in primary disorders and excluding environmental conditions.

More recently, the association of ApoB/ApoA1 ratio with future CV risk was demonstrated [29] in 396 children and adolescents and the predictive value of ApoB established alongside the changes occurring at different ages [28].

The relevance of the present study is as follows: first, it reports on ApoB concentrations in Italian children and adolescents, dyslipidemia affected or healthy; second, the strength of ApoB is related to the lipid profile as a tool to improve hyperlipidemia diagnosis; third, the number of participants is relevant, never tested before in the Italian population.

The limitation of the study is the lack of a final diagnosis for children and adolescents, and more defined cut-off levels should have been provided to refer to in medical practice. 

From this horizon, we conclude that the availability of ApoB levels represents an advantage as an important marker to distinguish between healthy or dyslipidemic children, to reach an early diagnosis of primary disorders, and hence to ameliorate health outcomes in young subjects. In any case, it is not negligible to note that, to optimize and limit the use of resources and costs, the ApoB dosage should be recommended as a second line of action.

## Figures and Tables

**Table 1:** Lipid profile, Apolipoprotein B and Anthropometric Measures of Partecipants.

Lipid profile and Apolipoprotein B of participants.							
		**Subjects** **(#)**		**Lipid mg/dL**	**Mean ApoB mg/dL**		
				
Total-C		411		000–170	65		*p* < 0.0001
		585		171–200	80		
		881		201–500	110		
				
LDL-C		618		≤110	68		*p* < 0.0001
		1259		>110	102		
				
HDL-C		687		≤50	94		See Table 4
		1189		>50	89		
				
Triglycerides		1552		≤110	89		See Table 5
		325		>110	100		

Anthropometric measurements of participants, gender and age-related.							
**F + M**		**Female**			**Male**		
**Age** **(y)**	**Pt** **(#)**	**Pt** **(#)**	**Height** **Mean ± S.D. (cm)**	**Weight** **Mean ± S.D. (kg)**	**Pt** **(#)**	**Height** **mean ± S.D. (cm)**	**Weight** **Mean ± S.D. (kg)**
1	14	3	85 ± 2	12 ± 1	11	81 ± 8	11 ± 2
2	38	19	92 ± 2	12 ± 1	19	94 ± 4	13 ± 1
3	58	37	100 ± 4	15 ± 4	21	98 ± 3	14 ± 1
4	80	49	107 ± 3	19 ± 4	31	107 ± 4	18 ± 3
5	100	49	113 ± 5	21 ± 6	51	114 ± 5	21 ± 5
6	133	82	119 ± 5	23 ± 8	51	120 ± 6	25 ± 6
7	148	76	128 ± 6	31 ± 9	72	125 ± 5	28 ± 6
8	208	117	133 ± 6	34 ± 8	91	131 ± 5	32 ± 9
9	208	124	138 ± 6	37 ± 13	84	139 ± 6	38 ± 8
10	186	96	143 ± 9	41 ± 9	90	144 ± 6	41 ± 9
11	179	90	150 ± 5	50 ± 13	89	148 ± 6	44 ± 10
12	165	72	153 ± 6	49 ± 10	93	157 ± 7	57 ± 16
13	131	75	158 ± 7	55 ± 10	56	161 ± 15	58 ± 18
14	82	40	160 ± 5	56 ± 13	42	166 ± 7	59 ± 15
15	60	35	162 ± 4	57 ± 13	25	173 ± 2	83 ± 14
16	43	27	160 ± 9	59 ± 19	16	171 ± 8	64 ± 12
17	34	15	164 ± 7	54 ± 8	19	174 ± 7	74 ± 10
18	10	4	164 ± 6	56 ± 9	6	175 ± 5	80 ± 9
total	1877	1010			867		

BMI of participants, gender and age-related							
**F + M**		**Female**				**Male**	
**Age** **(y)**	**Pt** **(#)**	**Pt** **(#)**	**BMI** **Mean ± S.D.** **(Ratio)**			**Pt** **(#)**	**BMI** **Mean ± S.D. (Ratio)**
1	14	3	17 ± 1			11	16 ± 1
2	38	19	14 ± 1			19	15 ± 1
3	58	37	15 ± 1			21	15 ± 1
4	80	49	16 ± 2			31	15 ± 2
5	100	49	17 ± 3			51	16 ± 3
6	133	82	16 ± 3			51	17 ± 3
7	148	76	19 ± 3			72	17 ± 3
8	208	117	19 ± 4			91	18 ± 4
9	208	124	19 ± 3			84	19 ± 3
10	186	96	19 ± 4			90	19 ± 3
11	179	90	22 ± 3			89	20 ± 4
12	165	72	20 ± 4			93	22 ± 5
13	131	75	22 ± 3			56	21 ± 4
14	82	40	22 ± 5			42	21 ± 4
15	60	35	22 ± 4			25	27 ± 5
16	43	27	21 ± 6			16	22 ± 5
17	34	15	20 ± 2			19	24 ± 3
18	10	4	21 ± 3			6	24 ± 4
total	1877	1010				867	

F = female; M = male; y = years; Pt = patients; # = number; BMI = body mass index; S.D. = standard deviation.

**Table 2:** Apolipoprotein B (mg/dL) by age, gender, and total cholesterol (mg/dL).

Age (Years) Pt (#)	Gender	Total Cholesterol mg/dL	Obs (#)	Mean ApoB mg/dL	S.D.	*p*
		000–170	33	62	11	
	F	171–200	48	84	12	*p* < 0.0001
		201–500	76	128	48	
	
(1,5)		000–170	32	65	11	
	M	171–200	38	81	11	*p* < 0.0001
(290)		201–500	63	123	36	
	
	F + M	000–170	65	64	11	*p* < 0.0001
		171–200	86	83	12	
		201–500	139	126	48	

		000–170	78	67	11	
	F	171–200	168	80	13	*p* < 0.0001
		201–500	250	108	27	
	
(6,10)		000–170	65	65	11	
	M	171–200	128	79	12	*p* < 0.0001
(884)		201–500	195	107	28	
	
		000–170	143	66	11	
	F + M	171–200	296	80	13	*p* < 0.0001
		201–500	445	108	27	

		000–170	46	62	12	
	F	171–200	78	78	11	*p* < 0.0001
		201–500	113	103	25	
	
(11,13)		000–170	76	65	14	
	M	171–200	61	80	12	*p* < 0.0001
(476)		201–500	102	106	22	
	
		000–170	122	64	13	
	F + M	171–200	139	79	12	*p* < 0.0001
		201–500	215	104	24	

	F	000–170	37	64	12	*p* < 0.0001
		171–200	33	78	10	
		201–500	51	112	22	
	
(14,18)	M	000–170	44	65	11	*p* < 0.0001
		171–200	31	80	10	
(227)		201–500	31	107	22	
						
	F + M	000–170	81	64	11	*p* < 0.0001
		171–200	64	79	10	
		201–500	82	110	22	

Pt = patients; # = number; Obs = evaluated subjects; ApoB = apolipoprotein B; S.D. = standard deviation; *p* = *p* value; F = female; M = male.

**Table 3:** Apolipoprotein B (mg/dL) by age, gender, and LDL cholesterol (mg/dL).

Age (Years) Pt (#)	Gender	LDL Cholesterol mg/dL	Obs (#)	Mean ApoB mg/dL	S.D.	*p*
(1,5) (290)	F	≤110	47	66	12	*p* < 0.0001
		>110	110	115	38	

	M	≤110	41	67	12	*p* < 0.0001
		>110	92	110	35	

	F + M	≤110	88	66	12	*p* < 0.0001
		>110	202	113	37	

(6,10) (884)	F	≤110	134	70	15	*p* < 0.0001
		>110	362	100	25	

	M	≤110	124	69	13	*p* < 0.0001
		>110	264	101	26	

	F + M	≤110	258	70	14	*p* < 0.0001
		>110	626	100	26	

(11,13) (476)	F	≤110	81	67	14	*p* < 0.0001
		>110	156	96	24	

	M	≤110	91	67	18	*p* < 0.0001
		>110	148	98	21	

	F + M	≤110	172	67	16	*p* < 0.0001
		>110	304	97	23	

(14,18) (227)	F	≤110	52	66	11	*p* < 0.0001
		>110	69	105	23	

	M	≤110	48	67	12	*p* < 0.0001
		>110	58	95	21	

	F + M	≤110	100	66	12	*p* < 0.0001
		>110	127	100	23	


Pt = patients; # = number; LDL = low density lipoprotein; Obs = evaluated subjects; ApoB = apolipoprotein B; S.D. = standard deviation; *p* = *p* value; F = female; M = male.

**Table 4:** Apolipoprotein B (mg/dL) by age, gender, and HDL cholesterol (mg/dL).

Age (Years) Pt (#)	Gender	HDL Cholesterol mg/dL	Obs (#)	Mean ApoB mg/dL	S.D.	*p*
(1,5) (290)	F	≤50	71	110	42	*p* < 0.01
		>50	86	93	36	

	M	≤50	52	100	38	n.s.
		>50	81	94	35	

	F + M	≤50	123	106	40	*p* < 0.01
		>50	167	94	36	

(6,10) (884)	F	≤50	170	95	25	*p* < 0.01
		>50	325	91	27	

	M	≤50	101	94	26	*p* < 0.05
		>50	287	89	28	

	F + M	≤50	271	95	25	*p* < 0.001
		>50	613	90	27	

(11,13) (476)	F	≤50	83	91	25	*p* < 0.05
		>50	154	84	25	

	M	≤50	103	86	25	n.s.
		>50	134	86	25	

	F + M	≤50	188	88	25	n.s.
		>50	288	85	25	

(14,18) (227)	F	≤50	45	91	32	n.s.
		>50	76	87	24	

	M	≤50	61	82	20	n.s.
		>50	44	82	26	

	F + M	≤50	106	86	26	n.s.
		>50	121	85	25	


Pt = patients; # = number; HDL = high density lipoprotein; Obs = evaluated subjects; ApoB = apolipoprotein B; S.D. = standard deviation; *p* = *p* value; F = female; M = male; n.s. = not significant.

**Table 5:** Apolipoprotein B (mg/dL) by age, gender, and triglycerides (mg/dL).

Age (Years) Pt (#)	Gender	Triglycerides mg/dL	Obs (#)	Mean ApoB mg/dL	S.D.	*p*
(1,5) (290)	F	≤110	133	97	38	*p* < 0.01
		>110	24	121	44	

	M	≤110	124	96	35	n.s.
		>110	9	106	50	

	F + M	≤110	257	97	36	*p* < 0.01
		>110	33	117	45	

(6,10) (884)	F	≤110	415	90	26	*p* < 0.0001
		>110	80	103	24	

	M	≤110	328	89	28	*p* < 0.001
		>110	61	100	24	

	F + M	≤110	743	90	27	*p* < 0.0001
		>110	141	101	24	

(11,13) (476)	F	≤110	177	84	25	*p* < 0.001
		>110	61	94	24	

	M	≤110	183	84	25	*p* < 0.01
		>110	55	93	22	

	F + M	≤110	360	84	25	*p* < 0.0001
		>110	116	94	23	

(14,18) (227)	F	≤110	104	86	26	*p* < 0.05
		>110	17	102	30	

	M	≤110	88	80	23	*p* < 0.05
		>110	18	90	19	

	F + M	≤110	192	83	25	*p* < 0.01
		>110	35	96	25	


Pt = patients; # = number; Obs = evaluated subjects; ApoB = apolipoprotein B; S.D. = standard deviation; *p* = *p* value; F = female; M = male.

## Data Availability

The original data used for the present study are anonymously stored by Dr Tarcisio Niglio.
